# GMean—a semi-supervised GRU and K-mean model for predicting the TF binding site

**DOI:** 10.1038/s41598-024-52933-4

**Published:** 2024-01-30

**Authors:** Chai Wen Chuah, Wanxian He, De-Shuang Huang

**Affiliations:** 1 Guangdong University of Science and Technology, Songsan Hu, Dongguang, 523070 Guangdong China; 2 Guangxi Academy of Sciences, 98 Daling Road, Nanning, 530007 Guangxi China; 3 Eastern Institute for Advanced Study, Eastern Institute of Technology, Tongxin Road No. 568, Ningbo, 315201 China

**Keywords:** Cell biology, Computational biology and bioinformatics, Mathematics and computing

## Abstract

The transcription factor binding site is a deoxyribonucleic acid sequence that binds to transcription factors. Transcription factors are proteins that regulate the transcription gene. Abnormal turnover of transcription factors can lead to uncontrolled cell growth. Therefore, discovering the relationships between transcription factors and deoxyribonucleic acid sequences is an important component of bioinformatics research. Numerous deep learning and machine learning language models have been developed to accomplish these tasks. Our goal in this work is to propose a GMean model for predicting unlabelled deoxyribonucleic acid sequences. The GMean model is a hybrid model with a combination of gated recurrent unit and K-mean clustering. The GMean model is developed in three phases. The labelled and unlabelled data are processed based on *k*-mers and tokenization. The labelled data is used for training. The unlabelled data are used for testing and prediction. The experimental data consists of deoxyribonucleic acid experimental of GM12878, K562 and HepG2. The experimental results show that GMean is feasible and effective in predicting deoxyribonucleic acid sequences, as the highest accuracy is 91.85% in predicting K562 and HepG2. This is followed by the prediction of the sequence between GM12878 and K562 with an accuracy of 89.13%. The lowest accuracy is the prediction of the sequence between HepG2 and GM12828, which is 88.80%.

## Introduction

Mining of deoxyribonucleic acid (DNA) motifs is a fundamental step in gene function research. It is used to discover transcription factor binding sites (TFBSs). A transcription factor (TF) is an activator that promotes the boost in a gene’s transcription. TF allows different cells in the body to function differently with the same genome. When TF turnover is abnormal, it can lead to uncontrolled cell growth, which is best known as cancer. Therefore, identifying these TFs is a critical task to understand gene regulation, which can greatly improve our understanding of disease-associated phenomena and thus promote precision in drug discovery^1,2^.

DNA is a unique list of genetic codes that consist of no particular order of random letters; adenine (A), cytosine (C), guanine (G), and thymine (T). For example, the little DNA chunks sequences “CCCCACCCGT” and “CCCCTCACCC” are similar in appearance but hold no meaning that can be easily read or understood by humans. These specific DNA sequences come from the K562 chronic myelogenous leukemia and GM12878 lymphoblastoid cell, respectively, based on experimental processes. There have been research efforts aimed at mining the DNA binding specificity, as the findings from these researches will contribute to a better understanding of DNA metabolism, transcriptional regulation, and the development of therapeutic drugs^3–5^.

In recent decades, researchers have proposed numerous methods that can model the relationship between gene expression and TFBSs in promoter regions. For example, generative algorithms^6,7^ and discriminative methods^8,9^. These experimental analyses are usually time-consuming^10,11^. The challenge in these methods is to identify sites or DNA sequence patterns that serve as binding sites for TFs. However, the sites or DNA sequence patterns are completely unknown. This can lead to false alarms in motif detection.

In recent years, deep learning methods have made significant advancements in motif mining. There are three commonly used types of deep learning methods: convolutional neural network (CNN)^12,13^, recurrent neural networks (RNN)^14–16^, and hybrid CNN-RNN^17^. These techniques have been enhanced to autonomously predict and recognize motifs, resulting in better predictions based on the provided data. It is important to note that this data is labeled. However, the creation of labeled data requires substantial resources, time, and effort^18^, which may not always be available in practical scenarios.

Therefore, this research considers a hybrid semi-supervised model (GMean) that requires labelled data for the training phase and unlabelled data for the testing phase, as well as the prediction phase. This may overcome the need for large annotated datasets. The labelled data and unlabelled data are substrings of length *k* contained in a biological sequence. This basic method is known as *k*-mer selection, which overcomes the problem of mutual orthogonality by considering the dependence between genomic patterns. Then, the *k*-mer selection is tokenized to create blocks of genomic vocabulary for the deep learning model and the machine learning language model. The deep learning model in GMean is a gated recurrent unit (GRU) used to learn and extract the “motif” of the genome. The machine learning language model in GMean is K-means, which is used to predict the unlabeled genome.

Finally, the performance of GMean in terms of accuracy, precision, recall, and F1-score is investigated using different combinations of deep learning models and machine learning language models.

The GMean model is used to predict data from lymphoblastoid cell (GM12878), HepG2 (a human liver cancer cell line), and K562 (a chronic myelogenous leukemia (CML)), obtained from the Encyclopedia of DNA Elements (ENCODE). GM12878 is generated by Epstein-Barr virus, which may cause infectious mononucleosis. HepG2 is an immortal cancer cell derived from liver tissue. K562 is an immortal myelogenous leukemia cell that may cause cancers of the blood cells.

The remainder of this paper is organized as follows: “Materials and methods” presents the experimental processes, which include data preprocessing, the deep learning algorithm and the machine learning language. “The proposed model (GMean)” shows the proposed model—GMean. The evaluation matrices are shown in “Performance metrics”. “Result and discussion” contains the experimental results and discussions. Finally, “Conclusion and future work” concludes the paper.

## Materials and methods

The dataset includes 16,000 labelled and 4000 unlabelled DNA sequences in this work. The dataset consists of two columns: one column contains DNA sequences which are strings composed of combinations of ‘A’, ‘C’, ‘G’ and ‘T’. The other column contains class labels, which are either 0 or 1. A label of class 0 represents GM12878, while a label of class 1 represents K562. The labelled DNA sequences are used for training during the learning phase. On the other hand, the unlabelled DNA sequencesare used for testing and predicting during the testing phase. The ratio of the dataset for the learning phase to the testing phase is 4:1. The DNA sequences used in this study include GM12878 lymphoblastoid cell, K562 chronic myelogenous leukemia cell (CML), and HepG2 hepatoblastoma, a human liver cancer cell line. These datasets are from Encode which has been processed and been provided in^12^.

### Data preprocessing

Preprocessing of the data consists of converting the DNA sequence into a uniform format so that the proposed model can be understood. The processes include *k*-mers and text tokenizer. Algorithm 1 and Table 1 show the *k*-mers process that divides the DNA sequence “CCTCCCGAGAGA” into *k* biological sequences of equal length^19,20^. There are three different *k*-mers: 2-mers, 3-mers and 4-mers, which generate 16, 64 and 256 different biological sequences, respectively. Different *k*-mers generate different tokens and therefore affect the performance of the language model in terms of accuracy, precision, recall and F1-score. In this study, a *k*-mers range between two and four was chosen. The rational for this range is that 1-mer does not provide a useful DNA sequence relationship, and the accuracy of prediction decreases after 4-mers.

**Figure:**
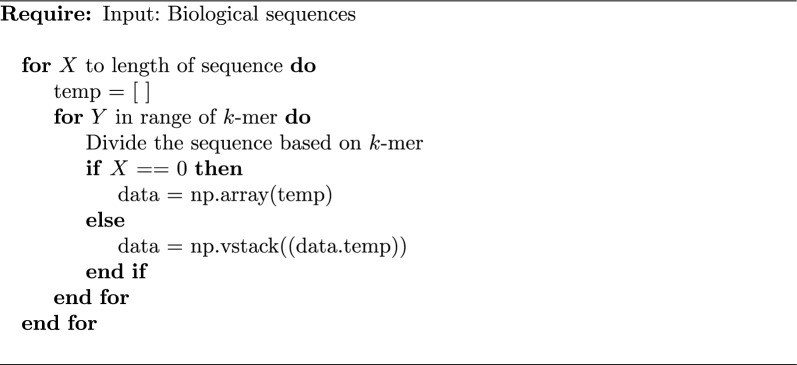
Pseudocode *k*-mer.

**Table 1:** Example of *k*-mers process..

*k*-mers	Biological sequences	Distinct sequence
2	CC CT TC CC CC CG GA AG GA AG GA	16
3	CCT CTC TCC CCC CCG CGA GAG AGA	64
4	CCTC CTCC TCCC CCCG CCGA CGAG GAGA	256

Tokenization converts the ‘character’ of a DNA sequence into a sequence of token. Tokenization is the process of preparing the token to enhance the performance analysis of the model. It consists of two majors processes: the first one is creating a bag of ‘vocabulary’, and the second one is converting the DNA sequence into integers based on the bag of ‘vocabulary’. For example, the bag of ‘vocabulary’ for 2-mers DNA sequence consists of 16 distinct sequence, such that ‘gg’: 1, ‘cc’: 2, ‘gc’: 3, ‘ct’: 4, ‘tg’: 5, ‘ag’: 6, ‘ca’: 7, ‘tc’: 8, ‘ga’: 9, ‘tt’: 10, ‘aa’: 11, ‘gt’: 12, ‘ac’: 13, ‘cg’: 14, ‘at’: 15, ‘ta’: 16. Next, it converts the long DNA sequence into integer based on the bag of ‘vocabulary’. For example, given the sequence “CCTCCCGAGAGA”, the tokenizing output based on 2-mers is (2, 4, 8, 2, 2, 14, 9, 6, 9, 6, 9).

### Gated recurrent unit

Gated recurrent unit (GRU)^21^ is a gating mechanism that is used in recurrent neural networks. It consists of an update gate, a reset gate, a candidate hidden state, and a hidden state, all of which work together to improve prediction accuracy. GRU is widely applied in addressing the vanishing gradient problem by utilizing the update gate and reset gate. The update gate, denoted as $z_t$, determines the amount of past knowledge that should flow into the memory. On the other hand, the reset gate, denoted as $r_t$, captures short-term dependencies and controls the flow of information out of the memory. The information considered in this process includes $h_{t-1}$ and $x_t$. The weight matrices for the update gate and reset gate are represented as $W_z$ and $W_r$, respectively. The bias vector for the update gate is denoted as $b_z$, while the bias vector for the reset gate is denoted as $b_r$. The activation function $\sigma$ is a sigmoid function, and the operation denoted as $+$ represents pointwise multiplication. The candidate hidden state, denoted as ${\hat{C}}_t$, is activated using the hyperbolic tangent function (*tanh*). The final hidden state is represented as $t_t$. The value of $z_t$ in the hidden state is either close to one or close to zero. If the value of $z_t$ is close to one, the old state is retained. Conversely, if the value of $z_t$ is close to zero, a new latent state of $t_t$ is generated. The gate structures and cell states are calculated according to the following equations^21^:

1 $$\begin{aligned} \begin{aligned}(b) {\textbf {Update gate}}: z_t&= \sigma (W_{z}x_t + V_{z}h_{h-1} + b_z) \\ {\textbf {Reset gate}}: r_t&= \sigma (W_{r}x_t + V_{r}h_{h-1} + b_r) \\ {\textbf {Candidate hidden state}}: {\hat{C}}_t&= tanh(W_{C}x_t + V_{C}(r_{t}. h_{t-1},x_t)) \\ {\textbf {Hidden state}}: t_t&= z_t.h_{t-1} + (1-z_t). {\hat{C}}_t \end{aligned} \end{aligned}$$

### K-means clustering

K-means (KM) is an unsupervised machine learning algorithm which is widely used in solving clustering problems. Equation (2)^22^ shows the process of K-means clustering by partitioning *i* cases into $b^{th}$ clusters, where $b=1,\ldots ,c$ and each case belongs to the cluster with the nearest mean.

2 $$\begin{aligned} \begin{aligned}{}[b] a_{b}=&\frac{\sum \nolimits _{i=1}^{n} {z_{ib} x_{ij}}}{\sum \nolimits _{i=1}^{n} {z_{ib}}}~\text{ and }\\ z_{ib}=&{ {\left\{ \begin{array}{ll} 1&{}\text{ if }~\left\| { {x_{i} -a_{b}} }\right\| ^{2}=\min \limits _{1\le b\le c} \left\| { {x_{i} -a_{b}} }\right\| ^{2} \\ 0, &{} \text {otherwise.} \\ \end{array}\right. }} \end{aligned} \end{aligned}$$

where *x* is the dataset, *c* is the number of clusters, $a_b$ represents the *b*th cluster, $z_{ib}$ is a binary variable indicating whether or not data point $x_i$ belongs to the *b*th cluster, and $|| x_{i}^{(j)} - a_b ||$ is the Euclidean distance between the data point $x_{i}^{(j)}$ and the cluster $a_b$. For this research, the number of clusters is set to two, as it is a semi-supervised model and there are two types of DNA sequences.

## The proposed model (GMean)

**Figure 1:**
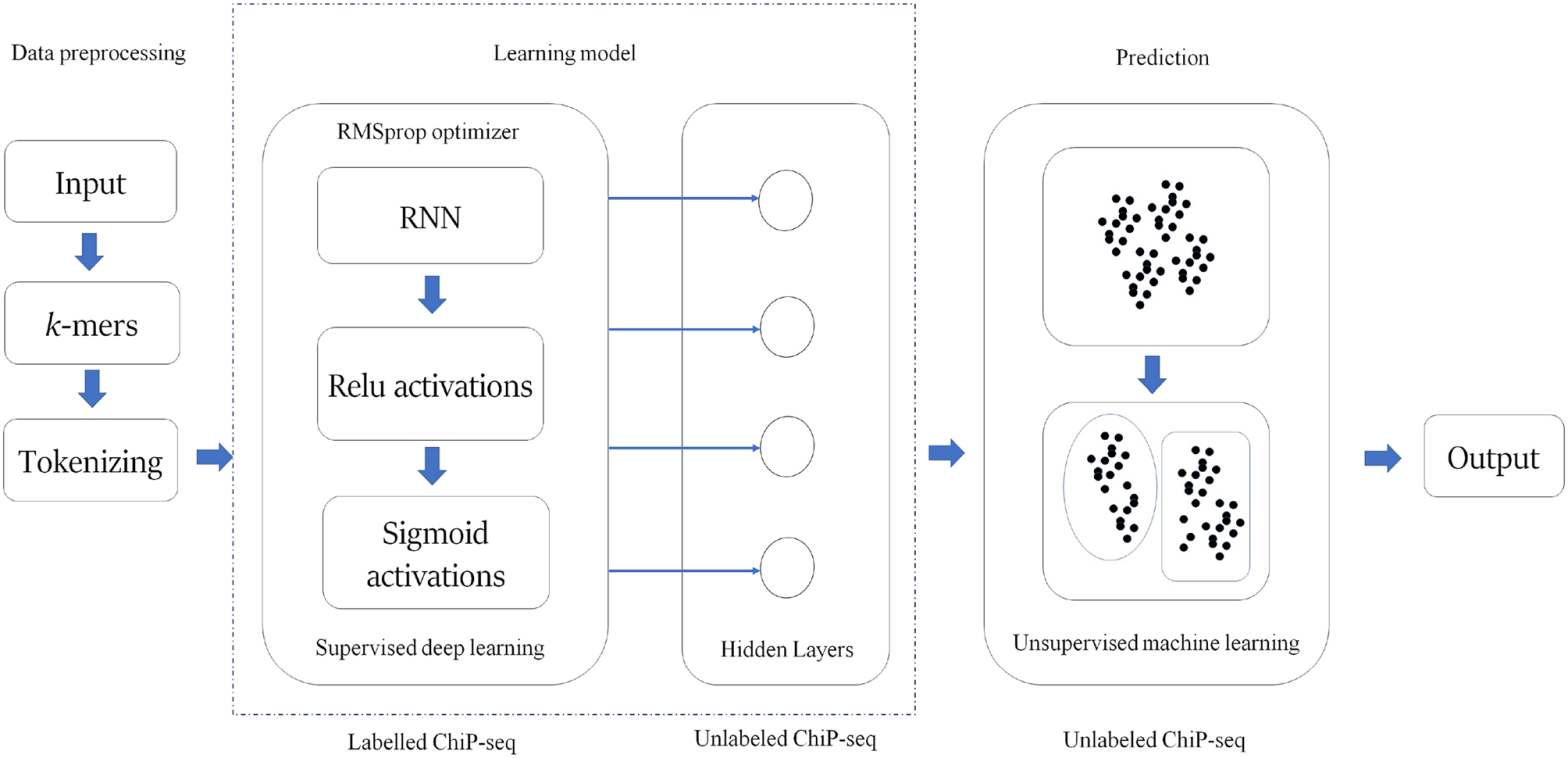
Proposed model—GMean.

The proposed model consists of three phases, there are data preprocessing phase, learning phase and prediction phase as shown in Fig. 1. The first phase of data preprocessing is *k*-mers tokenization, in which split the DNA sequence is split into *k*-mers and encoded into tokens. It is then mapped to a unique index. The *k* for this design is two.

**Figure 2:**
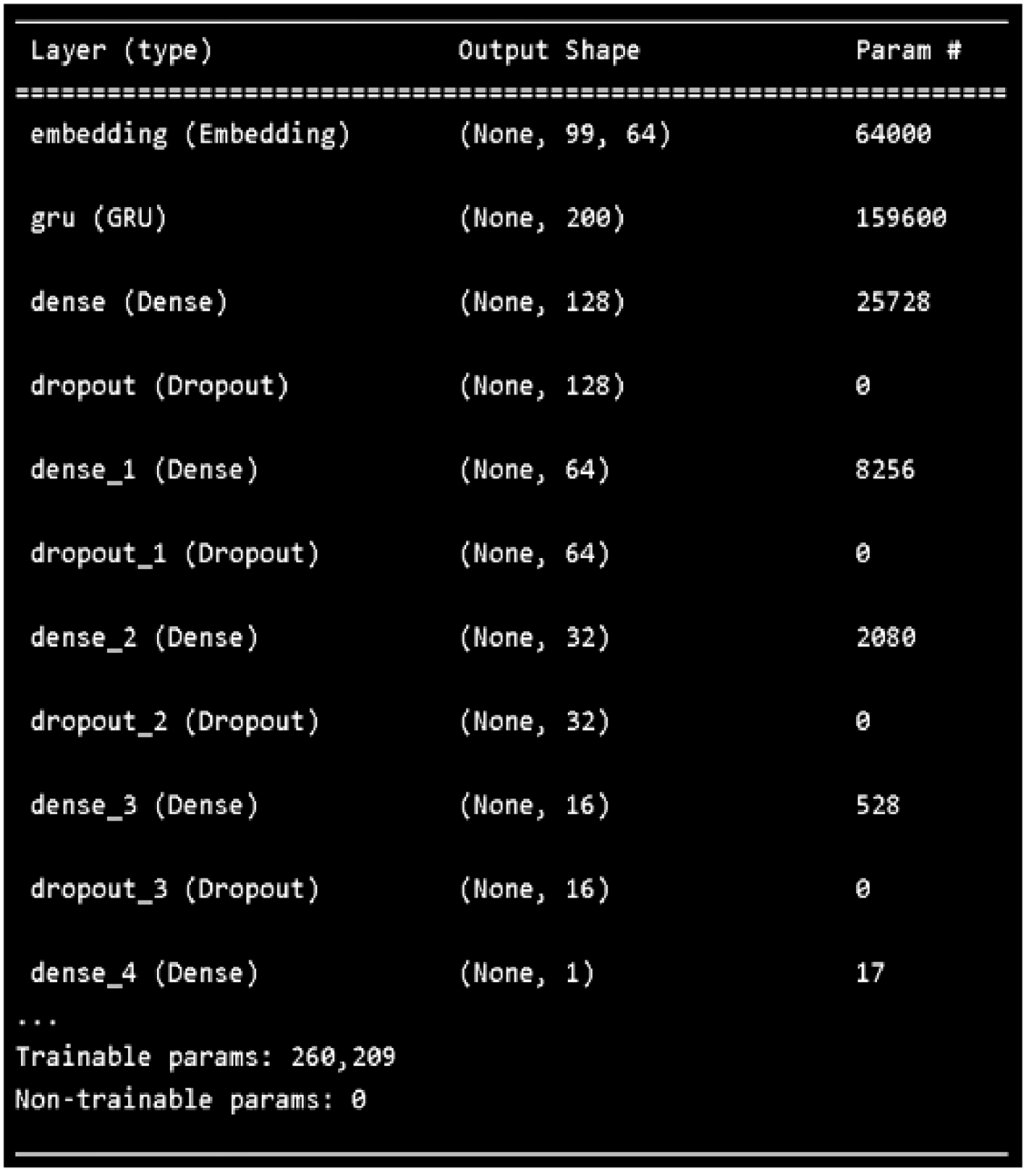
Supervised deep learning model summary.

The second phase is the learning phase. The supervised learning model consists of recurrent neural networks (RNNs) as shown in Fig. 2. The RNN in the GMean model consists of 200 GRU units. To force the GRU to learn the nonlinear behavior of the DNA sequence, the rectified linear units (relu) function and the sigmoid function are activated. The relu units are 128 units, 64 units, 32 units and 16 units. The unit of the sigmoid function is one. The GRU learns the relationships between the labelled tokenization DNA sequence by considering the context of the elements of its hidden state. The last block of the GRU makes the final decision on the probe’s remark. Relus are activated to eliminate the vanishing gradient problem during the learning process. The last layer is a non-linear transformation with sigmoid activation. The sigmoid activation produces a value between 0 and 1. This value represents the probability of a binding preference of each probe. The design for this model is simple, as it is based on the simple DNA sequence with only a list of combinations ‘A’, ‘C’, ‘G’ and ‘T’. During the learning process, overfitting may occur. Therefore, a dropout is added after the activation of each function. Now the supervised learning model is trained. To obtain the latent representation of the input learned by the model, we next create an unsupervised learning process that contains a sequential layer. We add the trained weights up to the fourth layer where the latent representation exists. The hidden representations of two classes of DNA sequences are generated.

The third phase is the prediction phase, where the above model is used. The unlabelled data is predicted as either a class 0 DNA sequence or a class 1 DNA sequence using K-means clustering (Eq. 2). K-mean calculates the distance between each data point in the DNA sequence cluster. The average of the data points in the cluster is calculated to find a specific center of the cluster. This results in the class 0 and class 1 clusters.

The proposed model is implemented based on Keras library. In the learning phase, the experiment randomly trains 12,800 labelled DNA sequences and validates 3200 labelled DNA sequences. There are 16,000 in total the labelled DNA sequences. Early stopping is applied in case of overfitting. Then, 4000 unlabelled DNA sequences are tested using the trained model. Finally, 4000 unlabelled DNA sequences are predicted either belong to class 0 or class 1. The performance for the models are evaluated. The model is simulated on graphical processing units (GPU) with Intel(R) Core (TM) i9-10980XE CPU@ 3.00 GHz, 128 GB random access memory and 1T hard disk.

## Performance metrics

A confusion metric with four elements (true positive, true negative, false positive, and false negative) is used to evaluate the performance of the models as shown in Table 2. The performance measures are accuracy (*P*), precision (*P*), recall (*R*) and F1-score (*F*1). True positive (TP) is predicted correctly and is actually positive. False positive (TP) is predicted correctly and is actually negative. True negative (TN) is predicted falsely and is actually negative. False negative (FN) is predicted falsely and is actually positive.

**Table 2:** Confusion matrix.

	Predicted	
Actual	True negative (TN)	False positive (FP)
	False negative (FN)	True positive (TP)

### Accuracy

Equation (3)^23^ formally defined the accuracy is the proportion of the number of correct predictions to the total number of predictions. Noted that the dataset for this simulation is a balance class. Hence, high accuracy provides high precision and high trueness.

3 $$\begin{aligned} \begin{aligned}(b)\; Accuracy (A)=\frac{TP + TN}{TP+FP+FN+TN} \end{aligned} \end{aligned}$$

### Precision

Precision refers to positive predictive value. As shown in Eq. (4)^23^, the precision is the fraction of correct predictions among the true and false positive (such as correct predict the DNA sequence is K562 DNA sequence). High precision requires high trueness.

4 $$\begin{aligned} \begin{aligned}(b)\;&Precision (P)=\frac{TP}{TP+FP} \end{aligned} \end{aligned}$$

### Recall

Recall refers to sensitivity of the model in capturing true positive value. As shown in Eq. (5)^23^, the recall value is the fraction of correct predictions among the actual positive value.

5 $$\begin{aligned} \begin{aligned}(b)\;&Recall (R)=\frac{TP}{TP+FN} \end{aligned} \end{aligned}$$

### F1-score

F1-score refers to seek for balance of the precision and recall. As shown in Eq. (6)^23^, F1-score measures is there any uneven class distribution.

6 $$\begin{aligned} \begin{aligned}(b)&F1-Score (F1)=\frac{2*P*R}{P+R} \end{aligned} \end{aligned}$$

## Result and discussion

The results and discussion consist of tables presenting performance metrics in percentages, which include accuracy, precision, recall, and F1-score. There are four ways to compare the performance metrics: (1) by comparing the performance metric for different units of GRU and *k*-mers data preprocessing; (2) by comparing the performance metrics of different learning models with the same prediction model; (3) by comparing the performance metrics of the same learning model with different machine learning models; and (4) by comparing the performance metrics of different types of datasets.

### Performance comparison with different number units of GRU and *k*-mers data preprocessing

Table 3 compares the performance metrics for different GRU units in the GMean model for genome assembly assessment. The DNA sequences used are GM12878 and K562, with 2-mers, 3-mers, and 4-mers. The number of GRU units tested are 100, 200, 300, and 400. The machine learning method used for these simulations is K-mean.

The results indicate that the GMean model performs best when using 2-mers and 200 GRU units. This combination achieves the highest accuracy in predicting whether a DNA sequence belongs to GM12878 or K562, with a percentage accuracy of 89.13%. The next best performance is achieved by using 100 GRU units, with a percentage accuracy of 86.20%. The sensitivity of the model using 3-mers and 200 GRU units captures the true positive values, with GM12878 having the highest percentage value of 96%.

Another notable trend observed in these simulation results is that the performance metric decreases as the number of GRU units increases. The worst performance is seen in the model that uses 2-mers and 400 GRU units, with only a 10% accuracy in predicting the DNA sequence. Therefore, the simulation is stopped when the number of GRU units reaches 400.

Based on these simulation results, the best performance in terms of accuracy, precision, recall, and F1-score is achieved by selecting 200 units of GRU and using 2-mers for data preprocessing in our proposed GMean model.

**Table 3:** Comparison results in term of accuracy, precision, recall and F1-score for different units GRU and *k*-mers data preprocessing in GMean model using dataset GM12878 and K562.

*k*-mers	Units	*A* (%)	Class	*P* (%)	*R* (%)	*F*1 (%)
2	100	86.20	0	81	94	87
			1	78	93	85
	200	89.13	0	88	91	89
			1	87	90	89
	300	88.76	0	86	93	89
			1	85	92	88
	400	10.40	0	10	11	10
			1	10	10	10
3	100	19.58	0	26	33	29
			1	6	8	7
	200	81.03	0	74	96	83
			1	66	94	78
	300	83.20	0	78	92	85
			1	74	91	81
	400	19.63	0	26	32	28
			1	7	10	8
4	100	67.03	0	69	62	65
			1	72	65	69
	200	67.65	0	65	78	71
			1	58	72	64
	300	32.65	0	29	25	27
			1	41	35	38
	400	41.25	0	18	5	8
			1	78	45	57

^a^Class 0 is GM12878. Class 1 is K562.

### Performance comparison with different learning models with fixed prediction model

Table 4 compares the performance metrics in terms of accuracy, precision, recall, and F1-score for four types of learning models along with a fixed machine learning model, namely K-mean. The learning models are GRU^21^, bidirectional GRU^14^(BiGRU), long short-term memory networks^24^ (LSTM) and bidirectional LSTM^25^(BiLSTM). Table 3 shows that the model with the 200 units performs the best, so all learning models are set with similar units. The experiments are conducted on the GM12878 and K562 datasets, which are processed using 2-mers.

The results in Table 4 indicate that the GRU learning model achieves the highest accuracy of 89.13% compared to the other models. The BiGRU learning model follows with an accuracy of 88.55%. On the other hand, the BiLSTM learning model exhibits the lowest accuracy of only 15.15%.

Furthermore, the LSTM learning model demonstrates the highest percentage in predicting positive values, with 92% for class 0 and 94% for class 1. It is followed by the BiGRU learning model. Conversely, the BiLSTM learning model performs the worst, with 10% accuracy for class 0 and only 2% for class 1.

Based on these simulation results, we select the GRU learning model for our proposed model GMean, as it achieves the best performance in terms of accuracy and recall. Additionally, the F1-score percentage is, on average, the second highest compared to the other learning models, at 89% for each class.

**Table 4:** Comparison results in term of accuracy, precision, recall and F1-score for different learning models with fixed prediction model.

Learning model	*A* (%)	Class	*P* (%)	*R* (%)	*F*1 (%)
GRU^21^	89.13	0	88	91	89
		1	87	90	89
BiGRU^14^	88.55	0	92	85	88
		1	92	86	90
LSTM^24^	84.80	0	92	76	83
		1	94	80	86
BiLSTM^25^	15.15	0	10	8	9
		1	2	19	21

^a^Class 0 is GM12878. Class 1 is K562.

### Performance comparison with same learning model and different machine learning models

Table 5 compares the performance metrics of the machine learning models in terms of GMean for predicting the DNA sequence in GM12878 or K562. The machine learning models evaluated are K-mean (KM), random forest regression (RF), support vector regression (SVR), and decision tree classifier (Tree). The learning model consists of 200 units of GRU, and the DNA sequence is tokenized with 2-mers.

The results indicate that the K-mean model outperforms other machine learning models in terms of accuracy, precision, recall, and F1-score. The highest accuracy achieved is 89.13%, while the decision tree classifier achieves an accuracy of 82.93% in predicting the DNA sequence. On the other hand, the random forest regression exhibits the lowest accuracy among the machine learning models, with only 50.13%. Based on these simulation results, the K-mean model is selected as the machine learning model in GMean.

**Table 5:** Comparison performance in term of accuracy, precision, recall and F1-score for different unsupervised machine learning language models.

ML	*A* (%)	Class	*P* (%)	*R* (%)	*F*1 (%)
KM^22^	89.13	0	88	91	89
		1	87	90	89
RF^26^	50.13	0	50	100	67
		1	1	100	1
SVR^27^	58.35	0	55	99	70
		1	18	94	30
Tree^28^	82.93	0	88	76	82
		1	90	79	84

^a^Class 0 is GM12878. Class 1 is K562.

### Performance comparison with different type of datasets

To further evaluate the performance of GMean, we perform experiments with different combinations of DNA sequences as shown in Table 6. The DNA sequences are GM12878, K562 and HepG2. There are three experiments in total.

**Table 6:** Comparison results in term of accuracy, precision, recall and F1-score for different dataset using GMean model.

Experiment	*A* (%)	DNA sequence	*P* (%)	*R* (%)	*F*1 (%)
1	89.13	GM12878	88	91	89
		K562	87	90	89
2	88.80	HepG2	91	87	89
		GM12878	91	87	89
3	91.85	K562	93	91	92
		HepG2	93	91	92

**Figure 3:**
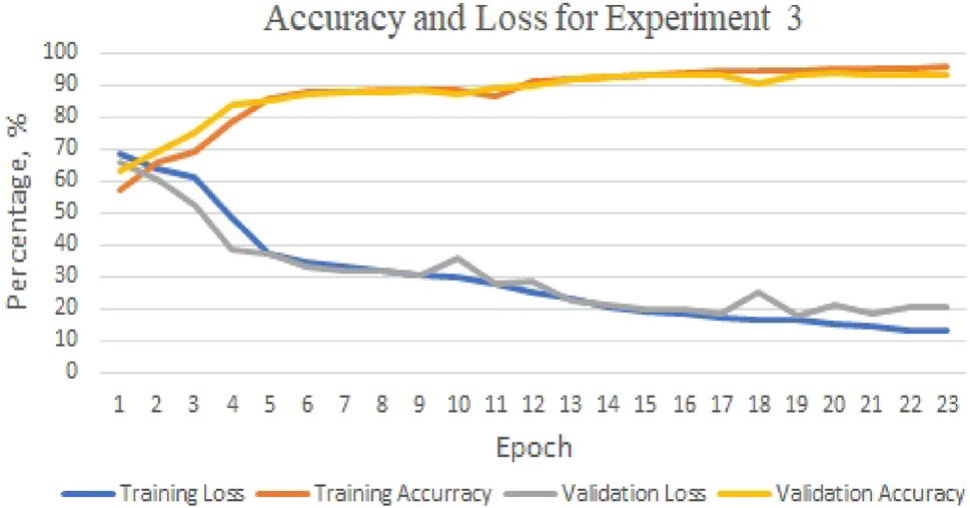
GMean model: the training/validation’s accuracy and loss for experiment 3.

The highest accuracy is obtained in Experiment 3 when predicting the DNA sequences K562 and HepG2, with a rate of 91.85%. The precision, recall, and F1-score for Experiment 3 are all above 90%. However, as shown in Fig. 3, the highest accuracy during the training phase and validation phase for Experiment 3 are 95.58% and 93.37%, respectively. This suggests the presence of overfitting.

**Figure 4:**
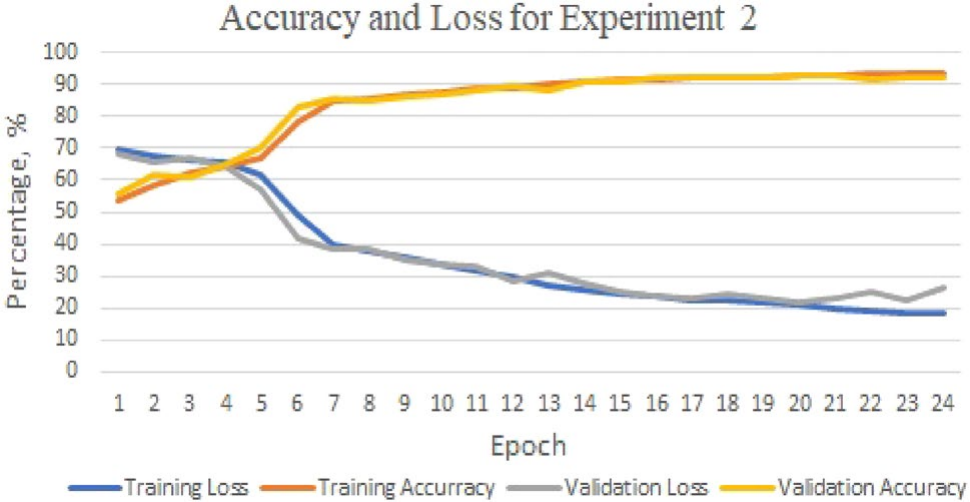
GMean model: the training/validation’s accuracy and loss for experiment 2.

As shown in Fig. 4, the lowest accuracy is recorded in experiment two. The accuracy is 88.80% for predicting the DNA sequences HepG2 and GM12878. The performance in predicting these DNA sequences, HepG2 and GM12878, in terms of precision, recall, and F1-score is consistent, with values of 91%, 87%, and 89%, respectively. Overfitting occurred as the highest accuracy during the training and validation phases were 93.46% and 92.12%, respectively”.

**Figure 5:**
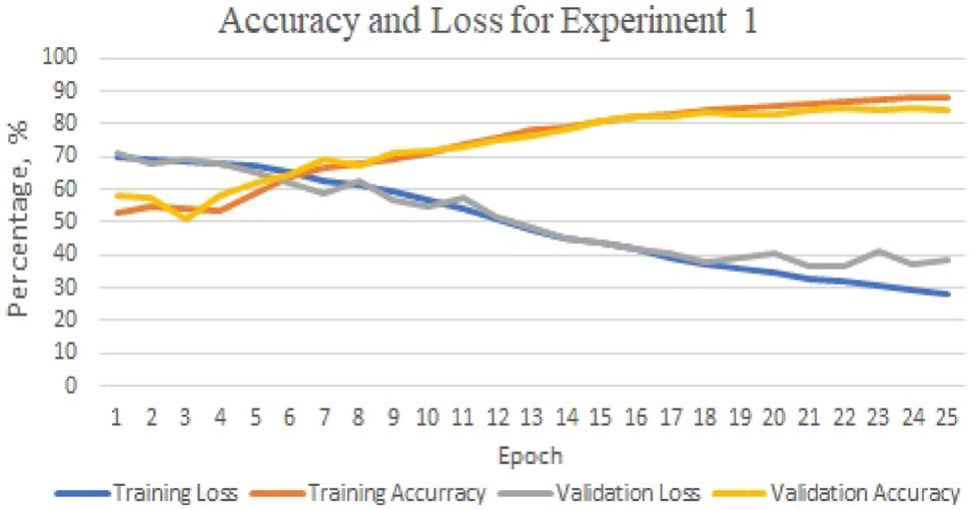
GMean model: the training/validation’s accuracy and loss for experiment 1.

Figure 5 shows the accuracy and loss for Experiment 1 during the training and validation phases, with the highest accuracy being 88.31% and 84.96%, respectively. The prediction accuracy is close to the training phase and is 89.13%.

In summary, the performance of the proposed GMean model in terms of accuracy is 89% on average for all three experiments. The results have shown that the GMean model is able to predict DNA sequences with an accuracy of over 80%. However, the GMean model still suffers from overfitting, and the proposed dropout in the model does not solve this problem.

## Conclusion and future work

The labelling of datasets is a common practice, but it can be costly and time-consuming. Acquiring labelled data can pose challenges due to these factors. However, employing a semi-supervised model can enhance performance even with limited labelled data. Hence, the focus of this research is on the hybrid semi-supervised deep learning and machine learning model, which we call GMean. GMean is proposed using a combination of GRU and K-Means. GMean is simulated and analyzed in terms of accuracy, precision, recall, and F1-score. The results show that the proposed 2-mers encoding GMean achieves an average accuracy of 89% compared to other combined deep learning and machine learning models. Although our model achieves better results, there is a limitation of GMean: overfitting. Therefore, there are ongoing research efforts to improve this model in order to maintain accuracy and reduce overfitting during the model learning phase”.

## Data Availability

The dataset for this research is taken from^12^ which available publicly at“https://github.com/jisraeli/DeepBind”.

## Code availability

The code for the current study are available from the corresponding author on reasonable request.
